# Genetic Modifiers of Thymic Selection and Central Tolerance in Type 1 Diabetes

**DOI:** 10.3389/fimmu.2022.889856

**Published:** 2022-04-07

**Authors:** Stephan Kissler

**Affiliations:** ^1^ Section for Immunobiology, Joslin Diabetes Center, Boston, MA, United States; ^2^ Department of Medicine, Harvard Medical School, Boston, MA, United States

**Keywords:** type 1 diabetes, Genome Wide Association Study (GWAS), thymic selection, autoimmunity, mouse model

## Abstract

Type 1 diabetes (T1D) is caused by the T cell-driven autoimmune destruction of insulin-producing cells in the pancreas. T1D served as the prototypical autoimmune disease for genome wide association studies (GWAS) after having already been the subject of many linkage and association studies prior to the development of GWAS technology. Of the many T1D-associated gene variants, a minority appear disease-specific, while most are shared with one or more other autoimmune condition. Shared disease variants suggest defects in fundamental aspects of immune tolerance. The first layer of protective tolerance induction is known as central tolerance and takes place during the thymic selection of T cells. In this article, we will review candidate genes for type 1 diabetes whose function implicates them in central tolerance. We will describe examples of gene variants that modify the function of T cells intrinsically and others that indirectly affect thymic selection. Overall, these insights will show that a significant component of the genetic risk for T1D – and autoimmunity in general – pertains to the earliest stages of tolerance induction, at a time when protective intervention may not be feasible.

## Introduction

Type 1 diabetes (T1D) is caused by the autoimmune destruction of pancreatic beta cells that produce insulin. The etiology of T1D has been investigated for more than 50 years (1). Animal models for autoimmune diabetes have been available for more than 40 years (2). And yet, the precise events that lead to beta cell autoimmunity remain incompletely understood. It is clear that T lymphocytes are key drivers of beta cell killing, as evidenced by genetic data, histological observations and mechanistic studies. However, a discrete trigger for beta cell autoimmunity, if it exists, is still being searched for. Environmental factors undoubtedly play a role in sensitizing individuals to type 1 diabetes. Both commensal microbes and viral infections have been implicated in diabetes etiology (3, 4). Not surprisingly, disease risk is also heavily modulated by genetic variants. The most prominent genetic risk factor for T1D is the highly polymorphic MHC region, driven by several high-risk HLA haplotypes (5, 6). In addition, a significant number of non-HLA genetic loci contribute to the heritable component of diabetes risk (7, 8). Linkage and association studies in the pre-genomic era uncovered the first non-HLA risk variant for T1D in the *Insulin* locus (9). This was followed a decade later by a risk variant in the *CTLA4* gene (10). In the early 2000’s, a handful of additional risk gene variants were discovered that included the *CD25*, *PTPN22* and *IFIH1* loci (11–13). In 2007, the results from the first genome wide association study (GWAS) conducted for an autoimmune disease revealed a much vaster landscape of risk variants for T1D across the genome (14). Subsequent GWAS with increasing statistical power have now brought the total of T1D-associated loci to more than 60 (7, 8, 15). Many GWAS for other autoimmune diseases followed the first T1D GWAS. A key insight from these association studies was that a large number of risk loci are shared between immune-mediated diseases. Only a minority of disease-associated genetic variants appear to be specific for T1D, while the majority seem to pertain more broadly to the risk of autoimmunity overall. This feature of shared genetic risk between diseases pointed to the fact that many disease variants impact basic immune regulatory mechanisms. Despite the enormous progress that GWAS have enabled in our understanding of disease genetics, it has been challenging to conclusively ascribe a function to individual disease variants. Notwithstanding, functional studies have highlighted the potential role of several T1D candidate genes in fundamental aspects of immune tolerance. This review will highlight several genes associated with the risk of T1D that impinge on the selection of the T cell repertoire in the thymus.

The development of T cells entails the migration of T cell progenitor cells from the bone-marrow into the thymic cortex, where the cells mature through several stages of CD4^-^CD8^-^ (double-negative or DN) thymocytes into CD4^+^CD8^+^ (double-positive or DP) cells. These DP thymocytes undergo a process of positive selection conditional on productive antigen-receptor interactions with thymic antigen presenting cells that include thymic epithelial cells (TECs). Positively selected cells further mature into single-positive (CD4SP or CD8SP) T cells that go on to migrate into the thymic medulla, the compartment where most of the negative selection takes place and that curates the T cell repertoire to eliminate highly self-reactive clones. Fully mature thymocytes that have undergone selection in the cortex and medulla then enter the circulation to become part of the immune surveillance machinery. The process of thymic selection that bars many, though not all, self-reactive clones from exiting the thymus is a key component of central tolerance – a protective quality control that occurs in a central location prior to mature T cells interacting with other cells throughout the body.

Defects in central tolerance lead to autoimmunity. In the most severe cases, single gene mutations can cause multiple immune pathologies. For example, mutations in the *FOXP3* gene prevent the induction of functional regulatory T cells in the thymus that are critical to the control of immunity. As a result, individuals with a mutant *FOXP3* allele develop the Immune dysregulation, Polyendocrinopathy, Enteropathy, X-linked (IPEX) syndrome that includes type 1 diabetes (16, 17). Another example is the *AIRE* gene whose disruption diminishes the expression of tissue-restricted antigens (TRAs) within the medullary thymic epithelium (18). TRA expression is necessary for the deletion of tissue-reactive T cell clones during thymic selection. Patients with deleterious *AIRE* mutations develop Autoimmune Polyendocrinopathy-Candidiasis-Ectodermal Dystrophy (APECED) that presents with multiple pathologies, often including type 1 diabetes (19, 20). The monogenic diseases IPEX and APECED are extreme examples of pathologies that arise as a consequence of defective central tolerance.

In this review, we will discuss more common gene variants associated with autoimmune disease including T1D. These common variants cause a much more subtle perturbation of central tolerance. However, even minor effects contribute to the overall risk of autoimmunity when compounded with other defects in immune tolerance.

## Genetic Modifiers of Thymocyte Function

Among the genes implicated in disease risk, several pertain to antigen receptor or cytokine receptor signaling. Changes in stimulatory cues that thymocytes receive during thymic selection significantly impact their developmental trajectory.

The first two examples of genes that modify thymocyte signaling encode the phosphatases PTPN2 and PTPN22. Both belong to the protein tyrosine phosphatase non-receptor family and impact key signaling events involved in the positive selection of thymocytes.

### PTPN2

The first GWAS for autoimmunity identified the single nucleotide polymorphism (SNP) *rs2542151* located 5.5kb upstream of the *PTPN2* on chromosome 18p11 (14, 21). A subsequent study further dissected this region and associated two intronic SNPs in the *PTPN2* gene with T1D (22). Both these SNPs are in strong linkage disequilibrium with *rs2542151*. Because *PTPN2* is the only gene in this region, it emerged as the strongest causal candidate for this particular disease association.

*PTPN2* encodes a phosphatase, and its expression is not restricted to immune cells. In fact, like many T1D-associated genes, PTPN2 is pleiotropic and affects the function of multiple cell populations including beta cells (23, 24). The phosphatase PTPN2 attenuates receptor signaling by desphosphorylating either receptors directly (e.g. InsR, EGFR), or their signaling transducers (e.g. SRC family kinases, JAKs, STATs). Most relevant to thymocyte development, PTPN2 decreases T cell receptor signaling *via* dephosphorylation of FYN and LCK, but also STAT5 phosphorylation that mediates IL-2 signaling.

Changes in PTPN2 function were shown to modify thymocyte development, positive selection and thymic lineage commitment of αβ TCR versus γδ TCR T cells (25, 26). Together, these effects have implications for the functionality of the T cell repertoire. Exactly how *PTPN2* variants skew T cells towards autoreactivity is difficult to dissect owing to the gene’s role in both thymocyte development, in the function of mature peripheral T cells and in the biology of multiple other cell populations relevant to T1D.

### PTPN22

*PTPN22* is another tyrosine phosphatase associated with T1D (12), but in this case, expression is restricted to lymphocytes. *PTPN22* encodes the lymphoid-associated phosphatase LYP that interacts with several mediators of antigen receptor signaling, including LCK, ZAP70 and TCRζ. Of interest, the genetic variant associated with T1D is located in the coding region of *PTPN22*. This is unusual because most disease-associated variations are intergenic or intronic, making it difficult to study their function. In contrast, the effects of the *PTPN22* disease-associated allele has been studied in more detail and have even been replicated in rodent models using genetic engineering.

The risk variant is a C to T substitution at position 1858 of the coding region, effecting an amino acid change in the protein sequence of LYP (R620W). This mutation has a direct implication for LYP function. The R620W substitution was shown to disrupt LYP’s interaction with a kinase, CSK, that negatively regulates phosphatase activity (12). This would be consistent with the first functional description of the risk variant that suggested a gain of function (27). The precise effect of the R620W mutation has been debated, however. Researchers modelled this mutation in mice by introducing an equivalent R619W substitution in PEP, the mouse ortholog of LYP (28). Data from this model initially suggested that mutant PEP was prone to faster degradation. This interpretation was later disputed and the preponderant hypothesis remains that the risk variant of *PTPN22* is a gain-of-function allele (29).

Additional studies in *Ptpn22* knockout and knockdown animals showed that the loss of PEP increased the frequency of regulatory T cells (Tregs) and suggested that animals were protected against autoimmunity (30, 31). These data were supported by the observation that the disease variant of *PTPN22* was associated with the frequency of circulating Tregs in human (32). Notably, *Ptpn22* deficiency increased the frequency of Tregs in the thymus (30), and this could relate to increased TCR signaling in the absence of the phosphatase that skews thymocytes towards a Treg transcriptional program. Extra-thymic effects of *Ptpn22* variation were also observed in both T and B lymphocytes, as could be expected given the phosphatase’s role in antigen-receptor signaling (29, 33). Ultimately, it is difficult to establish with certainty which immune cell population is most affected by *PTPN22* variation. Notwithstanding, the risk variant of *PTPN22* has a strong effect on thymic selection, with implications for the effectiveness of central tolerance.

### IL2RA

*IL2RA* encodes the high-affinity α chain of the IL-2 receptor and is also known as CD25. IL-2 signaling is critical to T cell development and function. Significantly, IL-2 is pivotal in the lineage commitment of Tregs in the thymus (34). Tregs are a key modifier of disease risk, and a target of experimental therapies for autoimmune disease. For example, low-dose IL-2 administration has been shown to expand Tregs in both humans and animal models (35, 36), where IL-2 therapy is a potent therapy of autoimmune diabetes.

*IL2RA* risk variants diminish IL-2 signaling (37, 38). This effect can be predicted to diminish both the development and maintenance of a functional Treg compartment that relies on IL-2 signaling both in the thymus and periphery. Complete IL-2 deficiency does not prevent T cell development (39) but causes severe inflammatory disease including colitis (40). While T1D-associated *IL2RA* variants lead to much more subtle changes in signaling, the gene has a central impact on immune regulation, starting with the generation of Tregs in the thymus.

### TAGAP

The signals that developing thymocytes receive and that direct their fate are tightly regulated. Part of this regulation relies on the spacial segregation of cues that guide positive selection versus negative selection. The first stages of thymocyte maturation occur in the thymic cortex. Once DP cells have been positively selected, they migrate into the thymic medulla to interact with a variety of antigen presenting cells that include medullary TECs (mTECs) presenting TRA for negative selection. The migration of thymocytes from the cortex to the medulla depends on both chemokines and adhesion molecules (41). *TAGAP*, the candidate gene for a genomic region associated with multiple immune diseases including T1D (15), plays a key role in releasing thymocytes from their cortical niche and allowing migration into the thymic medulla (42). This was demonstrated in a study of *Tagap* deficient mice, where thymocytes that recently underwent positive selection as measured by their expression of CD69 were retained in the thymic cortex. *Tagap* was found to mediate plexinD1 signaling that releases β1 integrin-dependent adhesion in the cortex (42). PlexinD1 is upregulated on the surface of positively selected thymocytes, allowing its ligand, sema3a, to facilitate chemotaxis towards the thymic medulla (43). A decrease in *TAGAP* expression diminishes the propensity of thymocytes to migrate from the cortex into the medulla. Longer dwell times in the cortex may allow maturation of the cells in an environment where they do not undergo the stringent negative selection imposed onto them in the medulla. This would lead to deficient tolerance induction by failing to delete autoreactive clones or to select Tregs that depend on interactions with medullary antigen presenting cells. Changes in the selection of thymocytes were observed in *Tagap* deficient mice, pointing to a role for this T1D risk gene in central tolerance (42).

The genes described above all relate to thymocyte-intrinsic pathways involved in the responsiveness to extracellular cues. Gene variants associated with T1D modify the sensitivity of thymocytes to TCR stimulation, cytokine stimulation and to chemotactic cues. Together, these effects can significantly redirect the fate of developing T cells with autoreactive potential and diminish either their deletion or their inclusion into the Treg compartment. Next, we will discuss disease risk genes that operate extrinsically by modifying the antigenic landscape that thymocytes navigate during selection.

## Genetic Modifiers of Antigen Presentation

A key function of the thymus is the selection of a TCR repertoire that is functional (through positive selection) and not harmful (through negative selection). Negative selection in particular relies on the presentation of antigens that T cell may encounter in various tissues (44). Many of these antigens are encoded by genes whose expression is restricted to specialized cell types. Most relevant for type 1 diabetes is insulin, one of a key antigens driving beta cell autoimmunity. More generally, tissue restricted antigens (TRAs) need to be presented within the thymus to allow central tolerance to take effect against these gene products. TRA presentation in the thymic medulla relies on three components. First, the antigen itself needs to be expressed within the thymus. Second, the antigen processing machinery needs to generate peptides from this antigen. Third, MHC molecules need to be present that are able to bind particular peptides so they can be presented on the cell surface. All three of these steps are subject to genetic control, as illustrated by T1D-associated genes discussed below.

### INS

A genomic region that included the insulin gene was the first non-HLA locus associated with T1D almost 30 years ago (9). The disease-associated haplotype encompasses a variable number of tandem repeats (VNTR) region 5’ of the *INS* gene. Initial analyses of the effect of the different VNTR alleles, termed class I, II and III based on their length, described a very small change in insulin expression associated with this polymorphism in fetal pancreas (45). How this subtle change would impact disease risk was unclear. Upon replication of this finding, Todd and colleagues speculated that the polymorphism may impact insulin expression in the thymus, rather than in the pancreas itself (46). Two studies published back to back in 1997 corroborated this hypothesis. The two independent papers reported that the protective haplotype that contains class III VNTR increased insulin expression in the thymus by 2-3 fold (47, 48).

In support of the hypothesis that VNTR alleles affected central tolerance, mouse models provided evidence that thymic expression of insulin had a strong effect on disease risk (49, 50). Unlike humans, mice harbor two insulin genes on separate chromosomes. While *Insulin 2* is expressed in both thymus and pancreas, *Insulin 1* is only expressed in beta cells. Deleting *Insulin 2* does not cause insulin insufficiency, because *Insulin 1* is fully functional and able to regulate glycemia on its own. However, in the absence of *Insulin 2*, thymic tolerance against insulin is severely impaired and the risk of diabetes is increased. While direct evidence in human for insulin’s role in central tolerance is lacking, it is also known that thymic *INS* expression is dependent on *AIRE* (51), whose deficiency leads to multiple pathologies that include T1D. Together, these observations support a key role for thymic insulin expression in establishing central tolerance to beta cell antigen.

### CLEC16A

TRA expression in the thymus is not sufficient in itself to ensure presentation of relevant peptides. Antigens need to be processed prior to being loaded onto MHC molecules for presentation on the surface of thymic epithelial cells or hematopoietic antigen presenting cells. One of the pathways involved in intracellular antigen processing and delivery to MHC compartments is autophagy (52). While MHC class I peptides are typically generated by the proteosome, MHC class II antigens rely on lysosomal degradation pathways. In this context, autophagy can shuttle endogenous proteins towards the lysosomal compartment for digestion and subsequent loading onto MHC class II molecules. The importance of autophagy for central tolerance had first been demonstrated by Klein and colleagues (53). TECs have remarkably high levels of constitutive autophagy. Disruption of autophagy in thymic epithelium caused multi-organ inflammation, indicative of defective central tolerance. This study demonstrated the importance of autophagy for antigen presentation in the thymic epithelium whose TRA expression is indispensable to central tolerance.

*CLEC16A* was shown to modulate autophagy, and the first indication of the gene’s function came from *Drosophila* studies where the *CLEC16A* ortholog *Ema* was implicated in the endolysosomal pathway (54), with subsequent data indicating a role in autophagy (55). Knockdown of *CLEC16A* was found to be highly protective in the NOD mouse model for type 1 diabetes (56). In this study, protection was not derived from *Clec16a* deficiency in immune cells but rather from gene knockdown in thymic epithelium. The loss of *Clec16a* diminished TEC autophagy and had repercussions for thymic selection and for the reactivity of the T cell repertoire (56).

*CLEC16A* is a prime example of a gene whose function is not obviously related to immune function. Yet, many cellular pathways contribute to robust thymic function that is critical for the establishment of central tolerance. It is likely that other T1D-associated genes whose function is not yet well characterized could affect immune tolerance in similarly unexpected ways.

### MHC Region

The final component of antigen presentation is the MHC molecule itself, encoded by HLA genes on Chromosome 6. When a TRA is expressed and processed into peptides suitable for MHC loading, the repertoire of peptides that are presented on the surface of thymic antigen presenting cells depends not only on the pool of peptides available but also from the binding preference of different HLA alleles. The HLA locus is the strongest genetic determinant for T1D risk (57). A handful of HLA haplotypes confer very high risk, while a few haplotypes are protective (58, 59). It is difficult to ascertain the stage at which HLA polymorphism most impacts pathogenesis, because MHC molecules are required throughout the lifetime of T lymphocytes. MHC/peptide complexes are required for T cell selection in the thymus, T cell maintenance in the periphery, and for the initiation of T cell responses in secondary lymphoid organs by antigen presenting cells bearing MHC class I and class II molecules.

The strongest T1D association in the HLA region derives from the HLA-DQ haplotype that encode MHC class II molecules. HLA-DQ2 (linked to HLA-DR3) and HLA-DQ8 (linked to HLA-DR4) are the most significant determinants of disease risk. Both haplotypes increase risk on their own, particularly in homozygous individuals. But their effect is even stronger in combination (when both HLA-DQ2 and -DQ8 are present) (60). This synergy is thought to be caused by trans-heterodimers, where the alpha and beta chains of the two different alleles (DQ2 and DQ8) are combined to form an alpha/beta heterodimer different from either DQ2 or DQ8 cis-heterodimers (61). One possible explanation for the high risk conferred by these particular HLA heterodimers is their preferential binding of peptides from beta cell antigens in a manner that is ineffective to enforce central tolerance yet sufficient to drive an immune response in the pancreas. Evidence for this mechanism lead to the hypothesis that peptide-HLA interactions in the low affinity range may be more likely to promote autoimmunity than high affinity binding peptides (62). Consistent with this notion, the T cell receptor of several CD4^+^ autoimmune T cell clones bind peptide-HLA complexes in unconventional, suboptimal conformations (63). While this weaker binding may derive in part from the TCR structure itself, the data support an overall model where the strength of interaction between autoreactive clones and their cognate peptide-HLA complexes is pivotal in bypassing negative selection. Therefore, the structure of HLA molecules, dictated by their genetic sequence, is central to the development of autoreactive clones in the thymus.

The same principles apply to MHC class I required for thymic selection of CD8^+^ T cells. Again, the structure of MHC class I molecules determines the pool of peptides that can be presented to developing thymocytes and the avidity of the TCR-MHC/peptide interactions at play during selection. MHC class I molecules are encoded by HLA-A, -B and -C genes. Rigorous analyses of the MHC region have shown that both HLA-A and HLA-B polymorphisms associate with the risk of T1D independently of the major effect of the MHC class II region (64). Subsequent experiments where the high-risk alleles HLA-B*39 or HLA-A*02 were expressed in transgenic mice devoid of endogenous MHC class I showed that these MHC alleles significantly changed the selection of the TCR repertoire (65). These data lend further support to a model where particular HLA alleles promote the thymic selection of an autoreactive repertoire prone to causing T1D.

## Conclusions

The list of disease-associated regions described in this brief review is not exhaustive, and other type 1 diabetes risk gene variants are likely to affect thymocyte selection by a variety of mechanisms. The examples cited above illustrate the wide range of mechanisms by which gene variations can modify T cell selection. Some risk genes operate cell intrinsically to desensitize thymocytes to negative selection or to diminish their likelihood of adopting a regulatory program. Other risk variant act extrinsically to shape the MHC/peptide landscape that fine-tunes the TCR repertoire and directs thymocytes to different selection trajectories. Most of these risk loci, whether they mediate intrinsic or extrinsic effects, have pleiotropic effects that can not only span the lifetime of a T cell but also alter the biology of other immune cell types. The result is a complex interplay of changes at many stages of immune function. To dissect individual components and to ascribe causal function to single gene variants remains exceedingly difficult and uncertain. Notwithstanding, our understanding of the genetics of autoimmunity and of T1D in particular have made great strides in the past 15 years. We now have a better grasp of the many fundamental changes in immune development and function that underlie autoimmunity. Defective central tolerance is almost certainly an important prerequisite for autoimmune diabetes, and one that is subject to genetic control by common variants.

When GWAS for T1D were first performed, they held much promise to yield new insight into disease etiology. In addition, there was hope that new knowledge of risk genes would lead to the rational design of novel interventions. This optimism has been significantly dampened by the realization that 1) identifying exact causal variants for disease-associated regions is often very difficult, 2) the precise functional contribution to pathogenesis of the many risk genes is still largely unresolved, and 3) pleiotropic effects of many causal variants would decrease the specificity of an intervention that targets these T1D risk genes. In the context of central tolerance, an additional challenge is that many of the changes described herein occur early in development, with long-lasting effects for immune function. Targeting thymic selection for disease prevention is possible in experimental models. This was shown by intrathymic islet transplantation in very young NOD mice (66, 67). However, this approach is unlikely to be effective at late pre-diabetic stages or in newly diagnosed patients, where islet autoimmunity is already established and ongoing. Notwithstanding, the development of an immune intervention for T1D based on disease genetics remains an enticing idea. Ultimately, only a better understanding of causal gene variants can help turn this idea into a testable clinical intervention.

## Author Contributions

SK planned and wrote the manuscript as a sole author.

## Funding

Research in the author’s laboratory is funded by the JDRF, National Institutes of Health (NIDDK, grants P30DK036836 and R01DK120445), Beatson Foundation and the Harvard Stem Cell Institute (HSCI).

## Conflict of Interest

The author declares that the research was conducted in the absence of any commercial or financial relationships that could be construed as a potential conflict of interest.

## Publisher’s Note

All claims expressed in this article are solely those of the authors and do not necessarily represent those of their affiliated organizations, or those of the publisher, the editors and the reviewers. Any product that may be evaluated in this article, or claim that may be made by its manufacturer, is not guaranteed or endorsed by the publisher.
